# Nutritional status among orphans and vulnerable children aged 6 to 59 months in Addis Ababa, Ethiopia: a community-based cross-sectional study

**DOI:** 10.1186/s40795-021-00431-5

**Published:** 2021-04-26

**Authors:** Nina Berr, Yemisrach Nigatu, Nebiyu Dereje

**Affiliations:** 1 Department of Medicine, Myungsung Medical College/Myungsung Christian Medical Center, Addis Ababa, Ethiopia; 2 Department of Public Health, Myungsung Medical College/Myungsung Christian Medical Center, P.O.Box 14972, Addis Ababa, Ethiopia

**Keywords:** HIV/AIDS, Stunting, Wasting, Underweight, Orphans, Vulnerable children

## Abstract

**Background:**

Childhood undernutrition is a global problem contributing to more than a third of under-five mortality. Orphans and vulnerable children (OVC) fare worse than children living with their parents. However, the nutritional and healthcare needs of OVC are under-recognized in Ethiopia.

**Methods:**

A community-based cross-sectional study was conducted among OVC aged 6 to 59 months. Multi-stage sampling technique was applied to select the households and eligible children included in the study (*n* = 584). An interviewer-administered questionnaire and anthropometric measurements were carried out. The proportions of stunting, wasting and underweight were determined based on the WHO Z-score cut-off. Factors associated with stunting were identified by Multivariable binary logistic regression analysis.

**Results:**

The prevalence of stunting, wasting and underweight were 35.1% (95% CI; 31.3–39.1%), 4.7% (95% CI; 3.2–6.7%) and 12.0% (95% CI; 9.6–14.9%), respectively. Stunting was significantly associated with initiation of complementary feeding after 12 months of age (AOR = 3.61; 95% CI 1.16–14.11), household food insecurity (AOR = 1.90; 95% CI 1.10–3.17), unplanned pregnancy (AOR = 1.90; 95% CI 1.03–3.42), age ≥ 2 years (AOR = 1.80; 95% CI 1.25–2.67), caretaker’s age ≤ 25 years (AOR = 1.50; 95% CI 1.03–2.16) and employment of the caretaker (AOR = 1.50; 95% CI 1.03–2.26).

**Conclusion:**

The prevalence of all forms of undernutrition among OVC was significantly higher than the national estimate that has been reported by consecutive Ethiopian Demographic and Health Surveys (EDHS). Policy makers and programmers working on nutritional interventions should give due emphasis to address the unmet need of OVC and focus on interventions which enhance household food security and caretaker’s awareness on child feeding and pregnancy planning.

**Supplementary Information:**

The online version contains supplementary material available at 10.1186/s40795-021-00431-5.

## Introduction

Undernutrition among children is a global problem hindering individuals and entire nations from achieving their full potential [1–3]. Globally, more than a third of under-five deaths are linked to undernutrition [4]. The attribution of HIV/AIDS for the child undernutrition is enormous [1–3].

Ethiopia has managed to achieve the targets set by Millennium Development Goals (MDG 4), namely reducing the under-five mortality rate by two-thirds [5, 6]. Moreover, Ethiopia has reduced the prevalence of undernutrition among under five children markedly over the years and is committed to reducing undernutrition further through the National Nutrition Strategy Programmes [7]. Nutrition is also firmly embedded as a priority in the country’s Health Sector Transformation Plan [8]. For instance, the prevalence of stunting, the predominant form of under nutrition, has been reduced to 25.4% in urban areas and only 14.6% in the capital Addis Ababa according to EDHS 2016 [7]. However, these figures are likely to rest on statistics which obscure the poverties endured by poorer urban children and the OVC, as Ethiopia is located in Sub-Saharan Africa, where the prevalence of HIV/AIDS and OVC are substantial [3, 4, 9]. Studies have shown that when child health statistics are unravelled, it is evident that even where services are nearby, children growing up in poor urban settings fare as badly as or worse than children living in rural poverty in terms of undernutrition and under-five mortality [4, 10].

An orphan or vulnerable child is a child who is at high risk of lacking adequate care and protection due to parental death, disease, disaster or acute poverty. The term orphan can be further defined as a child whose mother (maternal orphan), father (paternal orphan) or both (double orphan) have died [3, 11]. Researchers have long been intrigued by the question whether orphans and vulnerable children (OVC) living in the community suffer more undernutrition than non-OVC. The current literature on this matter shows conflicting results. Studies in various countries across the African continent including Kenya, the United Republic of Tanzania and Zimbabwe have shown that OVC are more undernourished than their counterparts. Particularly the proportion of stunting and underweight was markedly higher and OVC had overall poorer health outcomes [10, 12, 13]. On the other hand, an analysis of national survey data in Sub-Saharan Africa on under-five children in 40 countries found no differences in the nutritional status of OVC and non-OVC [14]. Therefore, the question of whether OVC are more undernourished than non-OVC remains unclear and seems to differ from one society to the other. Up to this day, there are only few studies in Ethiopia that look at the nutritional status of less fortunate children such as OVC and no such studies have been conducted in the capital city of Addis Ababa where the prevalence of HIV/AIDS and OVC is substantial. Hence, it is the aim of this study to bridge the gap by assessing the nutritional status and associated factors of stunting among OVC living in Addis Ababa city, Ethiopia.

## Methods

All methods were carried out in accordance with relevant guidelines and regulations.

### Study setting, design and population

A community-based cross-sectional study was conducted from May 01–31, 2019 in Addis Ketema sub-city, one of the ten sub-cities of Addis Ababa. It is a densely populated sub-city consisting of ten districts covering an area of only 8.64 km^2^ with a total population estimated at 320,000 as of 2017. The total number of OVC due to HIV and other causes were 20,655 in 2017 in the sub-city. OVC is a child who is at high risk of lacking adequate care and protection due to parental death, disease, disaster or acute poverty [3, 11].

All OVC aged 6 to 59 months, who had been living in the study area for a period of at least 6 months were eligible for inclusion and only the youngest child was selected per household. OVC who were severely ill or disabled were excluded due to the difficulty of obtaining accurate measurements. Also, OVC who were not found in three appointed interviews were excluded from the study and replaced by the next eligible OVC. Furthermore, children whose caretakers have a hearing difficulty preventing them from being interviewed were excluded.

### Sampling procedures

Sample size for the prevalence of stunting, wasting and underweight was determined by a single population proportion formula by taking prevalence of stunting (35.1%), the most common form of undernutrition which was taken from a study done among OVC in Hawassa town in 2016 [15], Z α/2 = 1.96, 5% margin of error, 10% non-response rate and 1.5 design effect. The calculated sample size was 584. The sample size for the analytical component (factors associated with stunting) was determined by a two population proportion formula by taking 80% power, 95% confidence interval (CI), 5% margin of error, proportion of stunting among literate care givers and proportion of stunting among illiterate care givers from the study conducted in Gondar [16]. The sample size calculated from this formula was 190. Thus, we took the largest possible sample size (*n* = 584) in our study.

A multi-stage sampling technique was employed to recruit study participants. In the first stage, out of the ten districts, three of them were selected randomly. Then, the total sample size was distributed to each district proportionally to the total households of the district. Social workers living in the community who are collaborating closely with the sub-city identified the households at risk wherein OVC reside in their respective villages of the selected districts. In order to facilitate the data collection, one social worker accompanied each data collector to the OVC households until the sample size was completed. In households with more than one child aged 6 to 59 months, only the youngest OVC was selected. Many of the households received financial support as part of the safety net program. However, none of the children received any form of nutritional support or supplement.

### Data collection procedure

Data were collected by using a structured standardized questionnaire (supplementary material 1 attached) and anthropometric measurements performed by the data collecting team with identical scales, measuring boards and MUAC tapes. The questionnaire was adapted from various sources including UNICEF [15, 17]. It has several contents including socio-demographic characteristics, housing and sanitation, feeding practices and dietary diversity, morbidity variables and household food insecurity. It was initially prepared in English and translated into Amharic language for data collection. Back translation of the questionnaire into English was carried out by an independent translator to check for the consistency of the translation. Moreover, the contents of the questionnaire was checked for cultural appropriateness and its content validity by senior experts (Nutrition and Public Health experts). Pre-test was also conducted in a district not included in our study to check for any inconsistencies and modified accordingly.

Data collection was facilitated by nurses and facilitators who were social workers familiar with the OVC in selected households. Fieldworkers were given training by the principal investigator on the objectives and methodology of the study, the contents of the questionnaire, the confidentiality of responses, the use of instruments and standard procedure of anthropometric measurement.

Height was measured in standing position for children ≥2 years and length was measured in recumbent position in children < 2 years. The child was barefooted and free of head wear. For measuring height the child was helped onto the baseboard with feet slightly apart. The back of the head, shoulder blades, buttocks, calves and heels were touching the vertical board. The assistant held the child’s knees and ankles. With the child’s chin held between thumb and forefinger and eyes facing directly forward, the interviewer pulled the headboard down to rest firmly on top of the child’s and read to the nearest completed 0.1 cm [18, 19]. For measuring length the child was placed on its back. The assistant standing opposite the tape held the child’s head against the headboard. The child’s eyes were looking straight up. The interviewer standing on the side of the measuring tape held down the child’s knees with the left hand and moved the footboard with the right hand flat against the soles. The measurement was read and recorded to the nearest completed 0.1 cm [19].

Weight was measured with the child lightly dressed on a standard scale and recorded to the nearest 0.1 kg. For children < 2 years of age, the caretaker was first weighed alone and again holding the undressed child. The difference between the two readings equalled the weight of the child. The scale was calibrated immediately before each session [19].

MUAC is the circumference of the undressed left upper-arm measured at the mid-point between the shoulder tip and elbow in children with a height > 65 cm. The interviewers bent the arm of the child at the elbow and identified and marked the olecranon and acromion processes as well as the midpoint between the two landmarks with a pen. Then, the arm was straightened and hung down the side of the body. The tape was placed around the arm at the marked mid-point at correct tape tension and the circumference was read to the nearest 0.1 cm and repeated twice to ensure accuracy. Colour coding indicates nutritional status [19].

### Ethical considerations

Ethical clearance was obtained from the Institutional Review Board (IRB) of Myungsung Medical College. The participants were informed about the objective of the study and written informed consent was gained from the caretakers. Illiterate caretakers were asked to sign the consent form after it was read to them by the interviewer. Moreover, the participants were at no risk of serious harm and had the right to decline participation or withdraw at any time during the interview. Caretakers of acutely malnourished children were urged to seek health care in a nearby facility. The information collected in the study will be treated confidentially and anonymity guaranteed by the principal investigator.

### Data management and analysis

Age was documented in completed months. If the caretaker was unsure of the child’s day of birth, the 15th day of the month was used and if the month of birth was unknown the midpoint of the year was used [15]. Food security was assessed using the Household Food Insecurity Access Scale (HFIAS) specifically adapted by the USAID Food and Nutrition Technical Assistance (FANTA) project for use in developing countries as a measure of the degree of food insecurity in the household in the past four weeks. Households were considered food-secure if they scored less than 17 and food-insecure if they scored ≥17 points [17]. Dietary Diversity Scores were calculated by adding the number of food groups consumed in the household over the 24-h recall period and graded as low (≤3) and high (≥ 4) based on the WHO designation of minimum dietary diversity if four or more food groups consumed in the last 24 h [15]. The prevalence of undernutrition was assessed by calculating the percentages of children who are stunted, wasted or underweight using ENA SMART based on the WHO − 2 Z-score cut-off and summarized by percentage and the respective 95% confidence interval. Bivariate and multi-variable binary logistic regression analyses were carried out to identify factors associated with stunting. Those variables with *ρ*-value < 0.25 in the bivariate analysis were considered for further multi-variable analysis and *ρ*-values of less than 0.05 were taken as a cut-off point for determining the significant association of independent variables with stunting. Odds ratio (OR) with 95% confidence interval was calculated to determine the strength of associations. Multicollinearity was checked by the multicollinearity diagnostics (Variance Inflation Factor (VIF) and the tolerance test). Goodness of model fitness was assessed by Hosmer Lemeshew goodness of fit test.

## Results

### Socio-demographic characteristics

The response rate of the study was 98.6%. Majority of the participants (52.9%) were female and ranged from 12 to 23 months in age (27.0%). On average, each household had 4.4 members and 1.25 children under the age of five. Among the participants, 13.8% were orphans. Most caretakers were married (69.2%) and close to one third (30.5%) were illiterate (unable to read and write). Over half of the households had a monthly income less than or equalling 1000 Ethiopian Birr (ETB), below the international poverty line of $1.90 per day [20]. The majority (87.3%) of households consisted of only a single room as living space (Table 1).

**Table 1:** Socio-demographic characteristics of the study participants in Addis Ketema sub-city, Addis Ababa, Ethiopia

Variables	Frequency	Percentage
**Age of the child (*n*** **= 575)**		
6–11 months	63	10.9
12–23 months	155	27.0
24–35 months	130	22.6
36–47 months	130	22.6
48–59 months	97	16.9
**Sex of the child (*n*** **= 561)**		
Male	264	47.1
Female	297	52.9
**Orphanage status (*n*** **= 574)**		
Non-orphan	495	86.2
Paternal orphan	62	10.9
Maternal orphan	11	1.9
Double orphan	6	1.0
**Number of household members (*n*** **= 570)**		
< 5	354	62.1
≥ 5	216	37.9
**Number of household members under age 5 years (*n*** **= 565)**		
< 2	441	78.1
≥ 2	124	21.9
**Relationship of caretaker with the child (*n*** **= 569)**		
Parent	531	93.3
Grand parent	26	4.6
Other	12	2.1
**Age of caretaker (*n*** **= 558)**		
≤ 20 years	42	7.5
21–30 years	352	63.1
31–40 years	134	24.0
> 40 years	30	5.4
**Sex of caretaker (*n*** **= 571)**		
Male	37	6.5
Female	534	93.5
**Educational status of caretaker (*n*** **= 573)**		
Unable to read and write	175	30.5
Able to read and write (no formal education)	29	5.1
Primary education	242	42.2
Secondary education	96	16.8
Above secondary education	31	5.4
**Occupation of caretaker (n = 573)**		
Housewife	216	37.7
Daily labourer	188	32.8
Unemployed	74	12.9
Other	95	16.6
**Marital status of caretaker (n = 571)**		
Single	47	8.2
Married	395	69.2
Divorced	85	14.9
Widowed	44	7.7
**Family monthly income (ETB) (*n*** **= 493)**		
≤ 600 (≤$20 USD)	121	24.5
601–1650 ($20 - $55 USD)	275	55.8
1651–3200 ($55–105 USD)	77	15.6
> 3200 (>$105 USD)	20	4.1

*ETB* Ethiopian Birr, *USD* United States Dollar

### Feeding practices and dietary diversity

In 86 % (86.1%) of children, the pregnancy was said to be planned and 95.9% mothers had antenatal care (ANC) follow up and delivered in a health facility. All of the mothers who have followed ANC have received iron and folic acid supplementation during their pregnancy. Almost 70 % of the children whose birthweight was known had a normal birthweight ranging from 2500 to 4000 g. Nearly all (92.8%) children were exclusively breastfed during the first six months but only a third (31.0%) were breastfed for the recommended 24 months. Vaccination coverage was practically universal at 98.1% but only around three quarters (73.4%) were said to have received complete vaccination for age or had taken Vitamin A in the last six months (70.9%). Four-fifths (79.2%) of household were considered food secure and 57.2% of children had a minimum dietary diversity while almost half (43.8%) had consumed less than four food groups in the last 24 h prior to the survey (Table 2). Two weeks prior to the survey, 24.4, 24.8, and 19.3% of the children had febrile illness, cough, and diarrhoea. However, only six children were known to be HIV positive.

**Table 2:** Feeding practices and dietary diversity among OVC, Addis Ketema Sub-city, Addis Ababa, Ethiopia

Variables	Frequency	Percentage(%)
**Planned pregnancy (*n*** **= 568)**		
Yes	489	86.1
No	79	13.9
**Antenatal care (ANC) follow up (n = 565)**		
Yes	542	95.9
No	23	4.1
**Place of birth (n = 570)**		
Health facility	545	95.6
Home	25	4.4
**Birth weight (*n*** **= 426)**		
< 2.5 g	106	24.9
2.5–4.0 g	293	68.8
> 4.0 g	27	6.3
**Was the child breastfed? (n = 573)**		
Yes	559	97.6
No	14	2.4
**Initiation of breastfeeding (*n*** **= 555)**		
First hour	546	98.4
After first hour	9	1.6
**Exclusive brestfeeding (*n*** **= 553)**		
Yes	513	92.8
No	40	7.2
**Total duration of breastfeeding in months (*n*** **= 300)**		
< 6	28	9.3
6 to 11	34	11.3
12 to 17	46	15.3
18 to 23	25	8.4
≥ 24	167	55.7
**Initiation of complementary feeding (*n*** **= 557)**		
At birth	21	3.8
Birth to 6 months	83	14.9
6 months to 12 months	438	78.6
after 12 months	15	2.7
**Type of first complementary food (*n*** **= 556)**		
Formula milk	156	28.0
Cow milk	106	19.1
Porridge	180	32.4
Adult food	114	20.5
**Method of complementary feeding(n = 556)**		
Hand	144	25.9
Cup and spoon	284	51.1
Bottle	128	23.0
**Dietary Diversity Score (*n*** **= 559)**		
Low (< 4)	239	42.8
High (≥4)	320	57.2

### Prevalence of stunting, wasting and underweight

**Fig. 1:**
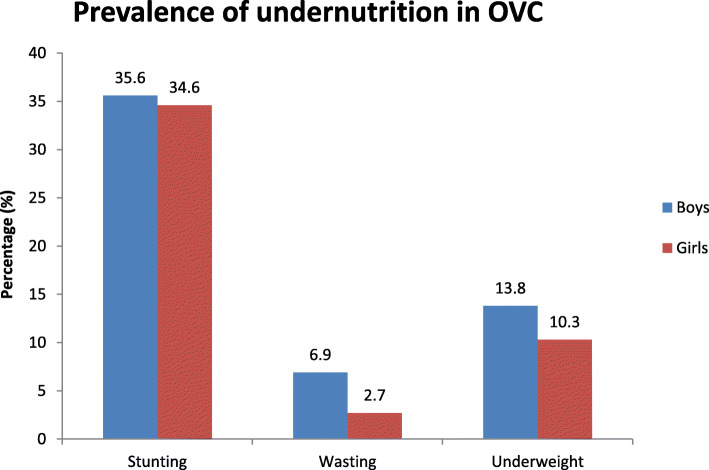
Prevalence of undernutrition among OVC in Addis Ketema Sub-city, Addis Ababa, Ethiopia

The prevalence of stunting, wasting and underweight were 35.1% (95% CI; 31.3–39.1%), 4.7% (95% CI; 3.2–6.7%) and 12.0% (95% CI; 9.6–14.9%) respectively in OVC living in Addis Ketema Sub-city, Addis Ababa. The distribution of the mal-nutrition by sex is given in Fig. 1.

### Factors associated with stunting

In the bivariate analysis (Supplementary material 2), age of the child, age of the care-taker, occupation of the care-taker, monthly income, planned pregnancy, maternal antenatal care follow up, breast feeding status, duration of breast feeding, place of birth, time of initiation of complementary feeding, household food insecurity were identified as candidate variables for further multi-variable analysis. However, in the further multi-variable analysis (Table 3), stunting was significantly associated with initiation of complementary feeding after 12 months of age, household food insecurity, unplanned pregnancy, age ≥ 2 years, caretaker’s age ≤ 25 years and employment of the caretaker. The odds of stunting among those OVC who initiate complementary feeding after 12 months of age was four times higher (AOR = 3.57; 95% CI 1.32, 7.62) as compared to those OVC who initiate complementary feeding within 6–12 months of age. The odds of stunting among those OVC with household food insecurity were two times higher (AOR = 1.86; 95% CI 1.10, 3.17) than those OVC with household food security. Similarly, the odds of stuning was two times higher among OVC age ≤ 23 months and care-taker’s age ≤ 25 years as compared to their counterparts. The odds of stunting was 1.5 times higher (AOR = 1.53; 95% CI 1.03, 2.26) among those OVC whose care-taker was employed as compared to those OVC whose care-taker was housewife.

**Table 3:** Factors associated with stunting among OVC in Addis Ketema sub-city, Addis Ababa, Ethiopia

Variables	Stunting		COR (95% CI)	AOR (95% CI)	***P*** value
	Yes N (%)	No N (%)			
**Age of the child**					
≤ 23 months	156 (41.7)	62 (31.0)	1.00	1.00	
24–59 months	218 (58.3)	138 (69.0)	1.60 (1.12, 2.40)	1.82 (1.25, 2.67)	0.004
**Age of the care-taker**					
≤ 25 years	114 (30.6)	79 (39.3)	1.47 (1.03, 2.11)	1.49 (1.03, 2.16)	0.026
> 25 years	259 (69.4)	122 (60.7)	1.00	1.00	
**Occupation of the care-taker**					
Housewife	154 (42.2)	62 (31.2)	1.00	1.00	
Employed	163 (44.7)	111 (55.8)	1.69 (1.16, 2.48)	1.53 (1.03, 2.26)	0.031
Unemployed	48 (13.2)	26 (13.1)	1.35 (0.77, 2.36)	1.35 (0.76, 2.40)	0.293
**Time of initiation of the complementary feeding**					
Birth to 6 months	74 (20.5)	29 (14.9)	0.70 (0.44, 1.12)	0.68 (0.31, 1.49)	0.353
6–12 months	281 (77.8)	157 (80.5)	1.00	1.00	
> 12 months	6 (1.7)	9 (4.6)	3.69 (1.36, 7.68)	3.57 (1.32, 7.62)	0.003
**Planned pregnancy**					
Yes	324 (88.5)	165 (82.5)	1.00	1.00	
No	42 (11.5)	36 (17.2)	1.68 (1.12, 2.86)	1.87 (1.13, 3.42)	0.032
**Household food insecurity**					
Secure	304 (81.9)	149 (74.1)	1.00	1.00	
Insecure	67 (18.1)	52,925.9)	1.58 (1.09, 2.45)	1.86 (1.10, 3.17)	0.015

## Discussion

The present study revealed that undernutrition among OVC less than five years of age is much higher than that was reported by Mini- EDHS 2019 at which the prevalence of stunting, wasting and underweight in Addis Ababa was 13.9, 2.3 and 4.7%, respectively [21]. Although there is an overall decreasing trend of undernutrition among children less than five years of age, the findings of this study revealed that among underprivileged children, the prevalence is still high. At 35.1 and 12.0%, the prevalence of stunting and underweight among OVC in Addis Ketema sub-city is more than 2.5 times higher than their counterparts living in Addis Ababa. Similarly, the prevalence of wasting (4.7%) is double that of their peers. This discrepancy could reflect that the EDHS might not represent the minority groups such as the OVC and may lead to underestimation of the prevalence of undernutrition in the city. Hence, the finding of this study calls for appropriate interventions and decision making to address the alarmingly high burden of undernutrition in this under-recognized group of children.

Although this study shows a high prevalence of stunting among OVC, it did not reveal that orphan-hood itself is significantly associated with stunting in the vulnerable children. Moreover, no significant association has been identified between the type of orphan (double, maternal, paternal) and chronic undernutrition. It should be noted however, that the proportion of orphans (13.4%) was relatively low and a greater percentage of orphans may have produced more results. This is in contrast to a study done in Hawassa and Dilla, Ethiopia, where about a quarter of the participants were orphans. It showed that children whose parents were not alive were more likely to be stunted [15, 22]. Also, a study in Zimbabwe in 2007 revealed that maternal and paternal orphans had statistically significantly heightened risks of stunting [10]. On the other hand, the results of a study published in Uganda in 2013 showed that there was no statistical difference in the prevalence of chronic undernutrition between orphans (17.0%) and non-orphans (17.2%) and is thus more consistent with the results of this study [23]. Therefore, this study concludes that while vulnerable children suffer more undernutrition than their care-takers, the lack of a parent is not a contributing factor in the population studied.

Consistent with the study conducted in Gondar, Ethiopia, this study revealed that children older than two years had significantly higher odds of stunting [16]. This could be explained by the lower socio-economic status of the care-takers to serve optimal meals for the children once they initiate foods. In the earlier ages, children often get the recommended optimal nutrition from breast feeding [2].

The strongest association with stunting was found in children in whom complementary feeding was initiated after 12 months of age. This was not demonstrated by other studies. Rather a study done in Hawassa that examined the type of first complementary food, found that children whose first food was porridge were more likely to be stunted as compared to those with milk as their first diet [15].

Children from households which were classified as food insecure based on the Household Food Insecurity Access Scale (HFIAS) had nearly doubled odds of being stunted. While the study in Hawassa showed identical trends it was not proven to be statistically significant [15]. Another study that was conducted in Kenya in 2010 examined household food security among orphans in the capital Nairobi. While orphans were much more vulnerable to food insecurity than non-orphans, orphans did not display a higher proportion of stunting and thus household food insecurity was not a significant determinant of stunting unlike in this study [13]. This discrepancy might be attributed by the differences in the socio-demographic status between the study participants.

Other interesting findings of this study include that children whose principal caretaker was employed as opposed to being a housewife had higher proportions of stunting, while other studies failed to identify such a relationship between caretaker employment and stunting. One likely explanation may be that children whose caretaker was housewife, had longer durations of breastfeeding. Other associated factors of stunting identified in this study include age of caretaker ≤25 years and unplanned pregnancy. These findings can be explained by the fact that younger care takers might not be interested in child care or may not have adequate knowledge towards child care.

This study is the first study to determine the prevalence of undernutrition among the orphans and vulnerable children in Addis Ababa using a community based design. However, the findings of the study might be affected by the recall and social-desirability bias of the care takers.

## Conclusions

The prevalence of stunting, wasting and underweight among the OVC was found to be significantly higher than their counterparts living in the capital city, Addis Ababa. Most importantly, this study uncovers that the positive health statistics which point towards decreases in under nutrition as evidenced by consecutive EDHS data do not accurately reflect the condition of the many underprivileged children living in the society. The findings of this study underscore the need for special consideration of OVC by policy makers and programmers during their planning for nutritional interventions, budget allocation and preparing different guidelines. Moreover, the community or caretakers of OVC should be educated about the increased possibility of undernutrition among the OVC and they should provide supports needed to address their needs.

## Supplementary Information

**Additional file 1.** Questionnaire.

**Additional file 2.** Table: Associated factors of stunting among OVC, Addis Ketema Subcity, AA, 2019.

## Data Availability

Data is available upon reasonable request from the corresponding author.

## Abbreviations

AIDS: Acquired Immunodeficiency Syndrome
AOR: Adjusted Odds Ratio
CI: Confidence Interval
EDHS: Ethiopian Demographic Health Survey
ENA: Emergency Nutrition Assessment
ETB: Ethiopian Birr
FANTA: Food and Nutrition Technical Assistance
HFIAS: Household Food Insecurity Access Scale
HIV: Human Immunodeficiency Virus
MUAC: Mid-Upper Arm Circumference
OVC: Orphans and Vulnerable Child
UNICEF: United Nations Children’s Fund
USAID: United States Agency for International Development
WHO: World Health Organization
