# Supplementary material for: Nutritional status among orphans and vulnerable children aged 6 to 59 months in Addis Ababa, Ethiopia: a community-based cross-sectional study
Source: BMC Nutr. 2021 Apr 26;7:24. doi: 10.1186/s40795-021-00431-5 (PMC8073948; doi:10.1186/s40795-021-00431-5)
Supplement: Supplementary file 1 — Additional file 1. Questionnaire. [file 40795_2021_431_MOESM1_ESM.pdf]

Nutritional status among orphans and vulnerable children aged 6 to 59 months in Addis Ababa, Ethiopia: A community-based cross-sectional study

Nina Berr[1], Yemisrach Nigatu[2], Nebiyu Dereje[3*]

1 Department of Medicine, Myungsung Medical College/Myungsung Christian Medical Center, Addis Ababa, Ethiopia

Email nina.berr@yahoo.de

2 Department of Public Health, Myungsung Medical College/Myungsung Christian Medical Center, Addis Ababa, Ethiopia

Email yemisrachmmc@gmail.com

3 Department of Public Health, Myungsung Medical College/Myungsung Christian Medical Center, Addis Ababa, Ethiopia

Email neba.jahovy@gmail.com

* Corresponding author:

Nebiyu Dereje

Addis Ababa, P.O.Box 14972, Ethiopia

Phone Number: +251978788638

Email: neba.jahovy@gmail.com

Questionnaire

Code number of questionnaire: _____________
Date of interview:_______________ (day/month /year)
Interviewer's name: ___________________ Interviewer's signature: ___________________
Outcome of interview:

1. Completed
2. Partially completed
3. Declined
4. Respondent not available

Supervisor's name ___________________ Signature _______________ Date ____________

Demographic and socioeconomic characteristics

Q. 101 Woreda of the child ____________
Q. 102 Kebele of the child_____________
Q. 103 Age of the child in completed months __________
Q. 104 Sex of the child

1. Male
2. Female

Q. 105 No of members in the HH _______
Q. 106 No of children under 5 in HH _____
Q. 107 Are the parents of the child alive?

1. Yes
0. No

If no, skip to Q. 109
Q. 108 If yes, who is alive?

1. Father
2. Mother
3. Both
4. Unknown

Q.109 Caretaker's relationship with child

1. Parent
2. Grandparent
3. Sibling
5. Neighbour
6. Other, specify: _______________

Q.110 Age of the caretaker in years______
Q.111 Sex of the caretaker

1. Male
2. Female

Q.112 Educational status of the caretaker

1. Not able to read and write
2. Able to read and write
3. Grade 1 – 8
4. Grade 9 – 10
5. Grade 10 + 2 & above
6. College/University

Q.113 Occupation of the caretaker

1. Housewife
2. Private employee

3. Government employee
4. Daily labourer
5. Merchant
6. Unemployed
7. Other, specify: ______________

Q.114 Marital status of the caretaker

1. Single
2. Married
3. Divorced
4. Widowed
5. Separated

If single, skip to Q 116.

Q.115 Occupation of the spouse/partner

1. Housewife
2. Private employee
3. Government employee
4. Daily labourer
5. Merchant
6. Unemployed
7. Other, specify: _____________

Q.116 What is the household's total monthly income in ETB? _____

Housing and Sanitation

Q. 201 Number of rooms in the household

1. 1
2. 2
3. 3
4. $\geq$ 4

Q.202 Do you have a separate kitchen?

1. Yes
0. No

Q. 203. If no, where do you cook?

1. Indoors
2. Outdoors

Q.204 Where is your source of water supply?

1. Tap
2. Public stand
3. Protected well
4. Other, Specify _____________

Q. 205 How far is the water source?

1. Less than 10 min
2. More than 10 min

Q.206 What is the HH daily water usage in liters? _________

Q.207 Container used for water storage

1. Pot
2. Jeri Can
3. Bucket
4. Other, specify: ______________

Q. 208 Does the drinking water storage container have a cover?

1. Yes

0. No

Q. 209 How is drinking water fetched from the storage container?

1. Pouring
2. Dipping

Q.210 Do you wash your hands with soap and water before feeding the child?

1. Yes
0. No

Q.211 Do you wash your hands with soap and water after using the toilet?

1. Yes
0. No

Q.212 Do you have a latrine?

1. Yes
0. No

If no skip to Q.215

Q.213 What type of latrine do you use?

1. Traditional pit latrine
2. Ventilated Improved latrine
3. Water carriage type
4. Other, specify: _______________

Q. 214 Ownership of the latrine

1. Private
2. Shared

Q.215 How is domestic waste disposed?

1. Open field
2. Pit
3. Municipality Service
4. Other, specify:_______________

Feeding practices and dietary diversity

Q. 301 Was the child breastfed?

1. Yes
2. No
3. Unknown

If not yes, skip to Q.305

Q.302 When was breastfeeding initiated?

1. Within first hour after delivery
2. Within the first day
3. After the first day
4. Unknown

Q.303 Was the child exclusively breastfeed for the first 6 months?

1. Yes
2. No
3. Unknown

Q.304 For how long was the child breastfed?

Q.305 Age at complementary feeding

1. Immediately after birth
2. Within 6 months
3. Within 6 to 12 months
4. ≥Twelve month

Q.306 First complementary food

1. Formula

2. Cow's milk
3. Adult food
4. Porridge
5. Other, specify: _____________

Q.307 What was used to feed the child?

1. Hand
2. Cup and spoon
3. Bottle
4. Unknown

For questions 308 - 319, enquire whether the child ate any of these foods yesterday during the day or night, whether at home or outside the home.

Q.308 Porridge, bread, rice, noodles or other foods made from grains (tef, maize, sorghum, millet, wheat, barely)

1. Yes
0. No

Q.309 Pumpkin, carrots, sweet potatoes, white potatoes or other roots or tubers

1. Yes
0. No

Q.310 Dark green leafy vegetables (kale, cabbage) or other vegetables

1. Yes
0. No

Q.311 Fruits like ripe mango, papaya, banana, avocado, orange

1. Yes
0. No

Q.312 Flesh meat (beef, lamb, goat, chicken) or organ meat (liver, kidney, heart)

1. Yes
0. No

Q.313 Eggs

1. Yes
0. No

Q.314 Fresh or dried fish

1. Yes
0. No

Q.315 Beans, peas, nuts, lentils or seeds

1. Yes
0. No

Q.316 Milk and milk products (cheese, yogurt or other milk products)

1. Yes
0. No

Q.317 Food with oil, fat or butter

1. Yes
0. No

Q.318 Sugary foods such as sweets, cake, biscuits or soft drinks

1. Yes
0. No

Q.319 Condiments for flavour such as spices (salt, black pepper), chillies, herbs

1. Yes
0. No

Morbidity variables

Q. 401 Was the pregnancy wanted?

1. Yes
0. No

Q.402 Has the mother ever attended ANC during her pregnancy with this child?

1. Yes
0. No

Q.403 Place of birth of this child

1. Home
2. Health facility
3. Other, specify: ____________

Q.404 Who attended the delivery?

1. TBA
2. Health personnel
3. Other, specify____________

Q. 405 What was the child's birth weight?

1. <2500 g
2. 2500 – 4000g
3. > 4000g
4. Unknown

Q. 406 What was the preceding birth interval?

1. <1 year
2. <2 years
3. >2 years

Q. 407 What is the birth order of the child?

1. First or second order
2. Third or fourth order
3. Fifth order or above

Q.408 Did the child receive vaccination?

1. Yes
2. No
3. Unknown

If not yes, skip to Q.411

Q.409 Does it have a vaccination card?

1. Yes
0. No

Q.410 What is the vaccination status?

1. Complete for age
2. Incomplete for age

Q.411 Has the child received vitamin A supplementation (in the last 6 months)?

1. Yes
0. No

Q.412 Has the child suffered from measles?

1. Yes
0. No

For questions 413 to 415 enquire whether the child had the symptoms in the past two weeks.

Q.413 Diarrhea

1. Yes
0. No

Q.414 Cough

1. Yes

0. No

Q.415 Fever

1. Yes
0. No

Food insecurity

For questions 501 to 509 enquire whether the HH has experienced the following in the past four weeks.

501. Did you worry that your HH would not have enough food?

1. Yes

0. No

501a. If yes, how often did this happen?

1. Rarely[1]
2. Sometimes[2]
3. Often[3]

502 Was any HH member not able to eat the kinds of foods they preferred because of a lack of resources?

1. Yes

0. No

502a. If yes, how often did this happen?

1. Rarely
2. Sometimes
3. Often

503. Did any HH member have to eat a limited variety of foods due to a lack of resources?

1. Yes

0. No

503a. If yes, how often did this happen?

1. Rarely
2. Sometimes
3. Often

504. Did any HH member have to eat some foods that they really did not want to eat because of a lack of resources to obtain other types of food?

1. Yes

0. No

504a. If yes, how often did this happen?

1. Rarely
2. Sometimes
3. Often

505. Did any HH member have to eat a smaller meal than they felt they needed because there was not enough food?

---

[1] once or twice in the past four weeks

[2] three to ten times in the past four weeks

[3] more than ten times in the past four weeks

1. Yes

0. No

505a. If yes, how often did this happen?
1. Rarely
2. Sometimes
3. Often

506. Did any HH member have to eat fewer meals in a day because there was not enough food?

1. Yes

0. No

506a. If yes, how often did this happen?
1. Rarely
2. Sometimes
3. Often

507. Was there ever no food to eat of any kind in your HH because of lack of resources to get food?
1. Yes

0. No

507a. If yes, how often did this happen?
1. Rarely
2. Sometimes
3. Often

508 Did any HH member go to sleep at night hungry because there was not enough food?
1. Yes

0. No

508a. If yes, how often did this happen?
1. Rarely
2. Sometimes
3. Often

509. Did any HH member go a whole day and night without eating anything because there was not enough food?
1. Yes

0. No

509a. If yes, how often did this happen?
1. Rarely
2. Sometimes
3. Often

Anthropometric Measurements

Q.601 Does the child have bilateral pitting edema on clinical examination?
1. Yes
0. No

If yes, skip to Q. 603

Q.602 Weight of child in kg ___________
Q.603 Height/Length of child in cm______
Q.604 MUAC of child in mm (if Ht>65cm) ___
