# Supplementary material for: Nutritional status among orphans and vulnerable children aged 6 to 59 months in Addis Ababa, Ethiopia: a community-based cross-sectional study
Source: BMC Nutr. 2021 Apr 26;7:24. doi: 10.1186/s40795-021-00431-5 (PMC8073948; doi:10.1186/s40795-021-00431-5)
Supplement: Supplementary file 2 — Additional file 2. Table: Associated factors of stunting among OVC, Addis Ketema Subcity, AA, 2019. [file 40795_2021_431_MOESM2_ESM.pdf]

Nutritional status among orphans and vulnerable children aged 6 to 59 months in Addis Ababa, Ethiopia: A community-based cross-sectional study

Nina Berr[1], Yemisrach Nigatu[2], Nebiyu Dereje[3*]

1 Department of Medicine, Myungsung Medical College/Myungsung Christian Medical Center, Addis Ababa, Ethiopia

Email nina.berr@yahoo.de

2 Department of Public Health, Myungsung Medical College/Myungsung Christian Medical Center, Addis Ababa, Ethiopia

Email yemisrachmmc@gmail.com

3 Department of Public Health, Myungsung Medical College/Myungsung Christian Medical Center, Addis Ababa, Ethiopia

Email neba.jahovy@gmail.com

[*] Corresponding author:

Nebiyu Dereje

Addis Ababa, P.O.Box 14972, Ethiopia

Phone Number: +251978788638

Email: neba.jahovy@gmail.com

Table: Associated factors of stunting among OVC, Addis Ketema Subcity, AA, 2019

Independent variable	Dependent variable (Stunting)		COR (95% CI)	P-value (Crude)	AOR (95%CI)	P-value (Adjusted)
	Frequency (%)					
	No	Yes				
**Age in completed months**						
6 to 23	156 (41.7)	62 (31.0)	1			
24 to 59	218 (58.3)	138 (69.0)	1.60 (1.12, 2.40)	0.012*	1.82 (1.25, 2.67)	0.004**
**Gender of the child**						
Male	168 (46.3)	95 (48.2)	1.08 (0.77, 1.55)	0.660		
Female	195 (53.7)	102 (51.8)	1			
**Number of household members**						
< 5	232 (62.2)	121 (60.2)	1			
≥ 5	141 (37.8)	80 (39.8)	1.09 (0.78, 1.57)	0.639		
**Number of HH members under age 5**						
1	291(78.0)	150 (74.6)	1			
≥ 2	82 (22.0)	51 (25.4)	1.21 (0.79, 1.73)	0.359		
**Orphan status**						
Yes	47 (12.6)	30 (15.1)	1.23(0.75, 2.01)	0.416		
No	325 (87.4)	169 (84.9)	1			
**Orphan type**						
Maternal	8 (17.8)	3 (10.7)	1			
Paternal	37 (82.2)	25 (89.3)	1.80 (0.42, 8.61)	0.412		
**Relationship of caretaker with child**						

Parent	343 (93.0)	187 (94.4)	1			
Other	26 (7.0)	11 (5.6)	0.78 (0.32, 1.45)	0.493		
**Age of caretaker in years**						
≤25	114 (30.6)	79 (39.3)	1.47 (1.03, 2.11)	0.034*	1.49 (1.03, 2.16)	0.026**
>25	259 (69.4)	122 (60.7)	1			
**Gender of caretaker**						
Female	347 (93.5)	186 (93.5)	1			
Male	24 (6.5)	13 (6.5)	1.01 (0.46, 2.01)	0.977		
**Educational status of caretaker**						
Illiterate	104 (27.9)	71 (35.3)	1.64 (1.01, 2.65)	0.047*	1.49 (0 .90, 2.48)	0.109
Primary	178 (47.7)	92 (45.8)	1.24 (0.79, 1.95)		1.23 (0.77, 1.96)	0.389
Secondary or higher	91 (24.4)	38 (18.9)	1			
**Occupation of caretaker**						
Housewife	154 (42.2)	62 (31.2)	1			
Employed	163 (44.7)	111 (55.8)	1.69 (1.16, 2.48)	0.025*	1.53 (1.03, 2.26)	0.031**
Unemployed	48 (13.2)	26 (13.1)	1.34 (0.77, 2.36)		1.35 (0.76, 2.40)	0.293
**Marital Status of caretaker**						
Single	113 (30.3)	66 (32.8)	1.12 (0.77, 1.62)	0.531		
Married	260 (69.7)	135 (67.2)	1			
**Occupation of caretaker's spouse**						
Employed	252 (86.3)	137 (87.3)	1			
Unemployed	40 (13.7)	20 (12.7)	1.09 (0.61, 2.12)	0.776		

**Monthly household income in ETB**						
< 1000	176 (47.2)	109 (54.2)	1.32 (0.95, 1.86)	0.107*	1.329 (0.927, 1.905)	0.116
≥ 1000	197 (52.8)	92 (45.8)	1			
**Availability of a separate kitchen**						
Yes	41 (11.1)	17 (8.5)	1			
No	327 (88.9)	182 (91.5)	0.74 (0.37, 1.31)	0.330		
**Place of cooking**						
Indoors	213 (61.7)	114 (60.3)	0.94 (0.64, 1.36)	0.747		
Outdoors	132 (38.3)	75 (39.7)	1			
**Water Source**						
Tap	286 (76.7)	147 (73.1)	1			
Other	87 (23.3)	54 (26.9)	1.21 (0.807, 1.85)	0.347		
**Distance of water source in min**						
< 10	210 (56.8)	107 (53.5)	1			
≥ 10	160 (43.2)	93 (46.5)	1.14 (0.80, 1.59)	0.455		
**Daily water usage in L**						
≤ 25	88 (23.6)	67 (33.3)	1.62 (1.12, 2.33)	0.012*	1.40 (0.94, 2.08)	0.105
> 25	285 (76.4)	134 (66.7)	1			
**Method of water fetching**						
Pouring	221 (59.9)	124 (62.0)	1			
Dipping	148 (40.1)	76 (38.0)	0.92 (0.66, 1.34)	0.623		

**Handwashing before feeding**						
Yes	349 (95.6)	185 (92.5)	1			
No	16 (4.4)	15 (7.5)	1.77 (0.80, 3.89)	0.120*	0.86 (0.23, 3.22)	0.805
**Handwashing after using latrine**						
Yes	353 (95.7)	185 (92.0)	1			
No	16 (4.3)	16 (8.0)	1.91 (0.91, 3.97)	0.073*	1.52 (0.42, 5.50)	0.556
**Availability of latrine**						
Yes	174 (47.0)	93 (46.3)	1			
No	196 (53.0)	108 (53.7)	1.03 (0.74, 1.44)	0.862		
**Type of latrine**						
Traditional	364 (98.9)	199 (99.5)	2.18 (0.26, 4.57)	0.474		
Improved	4 (1.1)	1 (0.5)	1			
**Was the child breastfed?**						
Yes	359 (96.8)	199 (99.0)	1			
No	12 (3.2)	2 (1.0)	0.30 (0.11, 1.04)	0.098*	0.30 (0.11, 1.01)	0.100
**Initiation of breastfeeding**						
First hour	351 (98.6)	195 (98.5)	1			
After first hour	5 (1.4)	3 (1.5)	1.08 (0.26, 4.89)	0.917		
**Exclusive breastfeeding for 6 months**						
Yes	327 (92.9)	186 (93.0)	1			
No	25 (7.1)	14 (7.0)	0.99 (0.46, 1.99)	0.968		
**Total duration of breastfeeding**						

<6 months	20 (11.0)	7 (5.9)	0.48 (0.19, 1.18)	0.179*	0.67 (0.21, 2.16)	0.520
6 to 11 months	23 (12.7)	11 (9.3)	0.66 (0.31, 1.42)		0.78 (0.36, 1.71)	0.533
≥12 months	138(76.2)	100(84.7)	1			
**Initiation of complementary feeding**						
Birth to 6 months	74 (20.5)	29 (14.9)	0.701 (0.438, 1.124)		0.675 (0.307, 1.489)	0.353
6 to 12 months	281 (77.8)	157 (80.5)	1			
>12 months	6 (1.7)	9 (4.6)	3.69 (1.36, 7.68)	0.003*	3.57 (1.32, 7.62)	0.003**
**Type of first complementary feeding**						
Milk	176 (48.8)	85 (43.8)	1			
Porridge	114 (31.6)	66 (34.0)	1.20 (0.81, 1.79)			
Adult food	71 (19.7)	43 (22.2)	1.25 (0.79, 1.98)	0.530		
**Method of complementary feeding**						
Cup and spoon	179 (49.9)	104 (51.3)	1			
Bottle	92 (25.6)	36 (18.4)	0.67 (0.43, 1.06)		0.61 (0.32, 1.14)	0.137
Hand	88 (24.5)	56 (28.6)	1.09 (0.72, 1.66)	0.138*	1.16 (0.64, 2.09)	0.613
**Dietary Diversity score**						
Low (<4)	143 (39.6)	96 (48.7)	1.45 (1.02, 2.06)	0.037*	1.49 (0.90, 2.46)	0.125
High (≥4)	218 (60.4)	101 (51.3)	1			
**Planned pregnancy**						
Yes	324 (88.5)	165 (82.5)	1			
No	42 (11.5)	36 (17.2)	1.68 (1.12, 2.86)	0.033*	1.87 (1.13, 3.42)	0.032**

**ANC follow up**						
Yes	353 (97.0)	189 (94.5)	1			
No	11 (3.0)	11 (5.5)	1.87 (0.77, 4.94)	0.146*	2.79 (0.80, 9.73)	0.103
**Place of birth**						
Health facility	356 (96.7)	188 (93.5)	1			
Home	12 (3.3)	13 (6.5)	2.05 (0.82, 4.95)	0.074*	1.37 (0.14, 13.22)	0.577
**Birth attendant**						
Health professional	355 (96.7)	188 (94.0)	1			
Traditional birth attendant	12 (3.3)	12 (6.0)	1.90 (0.80, 4.32)	0.123*	0.32 (0.02, 4.28)	0.229
**Birthweight in g**						
< 2,500	64 (22.5)	42 (30.0)	1.45 (0.91, 2.30)	0.206*	1.22 (0.75, 1.99)	0.449
2,500-4,000	201 (70.5)	91 (65.0)	1			
> 4,000	20 (7.0)	7 (5.0)	0.77 (0.32, 1.89)		0.68 (0.26, 1.77)	0.451
**Preceding birth interval**						
< 2years	120 (61.5)	67 (63.2)	0.93 (0.56, 1.55)	0.776		
≥ 2 years	75 (38.5)	39 (36.8)	1			
**Birth order**						
First or second	268 (73.8)	154 (77.4)	0.82 (0.55, 1.24)	0.351		
Third or more	95 (26.2)	45 (22.6)	1			
**Vaccination status**						
Vaccinated	364 (98.4)	196 (98.5)	1			
Not vaccinated	6 (1.6)	3 (1.5)	0.93 (0.22, 3.56)	0.917		

**Vitamin A taken in last 6 months**						
Yes	253 (70.5)	140 (29.5)	1			
No	106 (71.1)	57 (28.9)	0.97 (0.65, 1.39)	0.883		
**Household food insecurity**						
Secure	304 (81.9)	149 (74.1)	1			
Insecure	67 (18.1)	52 (25.9)	1.58 (1.09, 2.45)	0.028*	1.86 (1.10, 3.17)	0.015**
* P-value < 0.25 in the bivariate analysis		** P-value < 0.05 in the multivariate analysis				
